# A single hydrophobic cleft in the *Escherichia coli *processivity clamp is sufficient to support cell viability and DNA damage-induced mutagenesis *in vivo*

**DOI:** 10.1186/1471-2199-11-102

**Published:** 2010-12-29

**Authors:** Mark D Sutton, Jill M Duzen, Sarah K Scouten Ponticelli

**Affiliations:** 1 Department of Biochemistry, and Witebsky Center for Microbial Pathogenesis and Immunology, School of Medicine and Biomedical Sciences, University at Buffalo, State University of New York, 3435 Main Street, 140 Farber Hall, Buffalo, NY 14214, USA; 2 Department of Biochemistry, School of Medicine and Biomedical Sciences, University at Buffalo, State University of New York, 3435 Main Street, 140 Farber Hall, Buffalo, NY 14214, USA; 3 Current Address: Department of Immunology, Roswell Park Cancer Institute, Buffalo, NY 14263, USA

## Abstract

**Background:**

The ubiquitous family of DnaN sliding processivity clamp proteins plays essential roles in DNA replication, DNA repair, and cell cycle progression, in part by managing the actions of the different proteins involved in these processes. Interactions of the homodimeric *Escherichia coli *β clamp with its known partners involves multiple surfaces, including a hydrophobic cleft located near the C-terminus of each clamp protomer.

**Results:**

A mutant *E. coli *β clamp protein lacking a functional hydrophobic cleft (β^C^) complemented the temperature sensitive growth phenotype of a strain bearing the *dnaN159 *allele, which encodes a thermolabile mutant clamp protein (β159). Complementation was conferred by a β^C^/β159 heterodimer, and was observed only in the absence of the *dinB *gene, which encodes DNA polymerase IV (Pol IV). Furthermore, the complemented strain was proficient for *umuDC *(Pol V) -dependent ultraviolet light (UV) -induced mutagenesis.

**Conclusions:**

Our results suggest that a single cleft in the homodimeric *E. coli *β sliding clamp protein is sufficient to support both cell viability, as well as Pol III, Pol IV, and Pol V function *in vivo*. These findings provide further support for a model in which different Pols switch places with each other on DNA using a single cleft in the clamp.

## Background

Viability of all organisms depends upon a capacity to both accurately repair damaged DNA, as well as tolerate DNA lesions that for whatever reason evade repair [1]. In contrast to repair, which acts to either directly reverse the damage, or to excise modified bases so that the affected sequence may be re-synthesized, DNA damage tolerance mechanisms act to enable replication past the damaged site, without catalyzing repair of the lesion(s). Generally speaking, DNA damage tolerance mechanisms fall into one of two broad classes: (i) daughter strand switching, which refers to a collection of recombinational mechanisms that act to physically restructure the DNA at the replication fork to enable the complementary daughter strand to act as template to support replication beyond the damaged site(s) [1,2]; and (ii) translesion DNA synthesis (TLS), which refers to the process by which one or more specialized DNA polymerases (Pols) are recruited to catalyze replication past damaged sites in the DNA [3]. Since most Pols capable of catalyzing TLS display remarkably low fidelity on undamaged DNA, their actions must be very tightly controlled *in vivo *to guard against unwanted mutations [4,5]. Although multiple mechanisms likely contribute to the coordinate regulation of replicative and TLS Pols, considerable effort over the past decade has been devoted to understanding the roles played in this process by the ubiquitous family of DnaN sliding clamp proteins [5,6].

**Figure 1:**
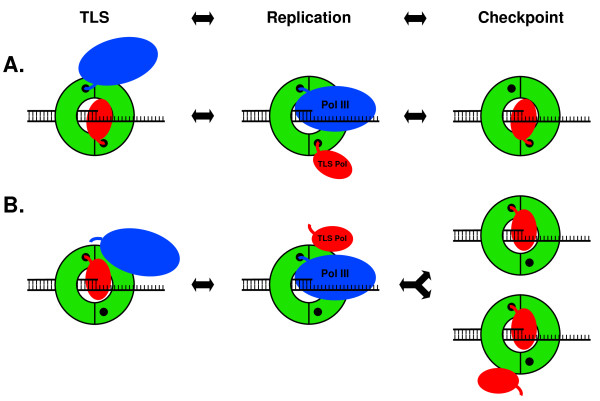
**Current models for Pol switching**. **(A) **The 'toolbelt' model posits that both a replicative and a TLS Pol associate with a single clamp, with each Pol binding to a separate cleft. In this model, the clamp acts to regulate sequential access of the two Pols to the DNA [9,10]. Displacement of Pol III from the clamp by Pol II, Pol IV, or Pol V is suggested to arrest replication in response to replication blocking DNA damage *via *a primitive DNA damage checkpoint control [18,20,38]. **(B) **In an alternative model, surfaces in addition to the cleft are postulated to play an important role in controlling access of the TLS Pol to the DNA (Residues E93 and L98 of the clamp control access of Pol IV [13,14], and possibly Pol V [16], for TLS), such that a single cleft in the clamp is sufficient to coordinate the switch (reviewed in [5]). TLS Pols may subsequently displace Pol III from the clamp to enable the checkpoint. Alternatively, binding of two or more TLS Pols may be required to displace Pol III and enable the checkpoint. See text for further details regarding these and other models. The green ring represents the β clamp, the small black circle in each clamp protomer represents the cleft, DNA is depicted in stick form, the blue oval represents Pol III, the red oval represents a TLS Pol, and the blue and red curved lines protruding from the respective Pols represents their CBMs, which must contact the clamp cleft to enable access of each respective Pol to the DNA.

**Figure 2:**
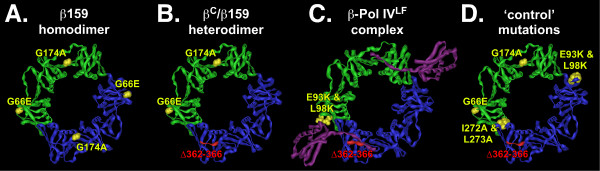
**Sliding clamp mutations utilized in this study**. **(A) **Positions of mutations present in β159 (G66E and G174A) are represented as yellow space filled atoms on the structure of the wild-type clamp. **(B) **Proposed structure of the β^C^/β159 heterodimer. Positions of G66E and G174A substitutions in β159 are represented as yellow space filled atoms, while residues 362-366, which are deleted from β^C^, are colored red. **(C) **Structure of the Pol IV little finger (Pol IV^LF^) domain in complex with the β clamp as reported by Bunting *et al. *[14]. Pol IV^LF ^is in purple. Pol IVLF contacts residues E93 and L98, as well as 362-366 of the clamp; position of E93K and L98K substitutions (yellow), and Δ362-366 (red) are indicated. **(D) **Positions of the E93K-L98K and I272A-L273A mutations used to characterize the ability of β^C ^to complement the temperature sensitive growth phenotype of the *dnaN159 *strain are shown on the presumed structure of the β^C^/β159 heterodimer, which bears the G66E, G174A, and Δ362-366 mutations. Figures were generated using Imol, and the coordinates for either the wild-type clamp (2POL), or the β clamp-Pol IV little finger complex (1UNN) obtained from the PDB.

Bacterial sliding clamps, termed β or DnaN, are encoded by the *dnaN *gene, and function as homodimers. Like their eukaryotic counterparts, these clamps are loaded onto DNA in an ATP-dependent manner by a multi-subunit clamp loader complex [7]. Once loaded, they recruit the replicative Pol (Pol III), as well as other partner proteins involved in various aspects of DNA replication, repair, and damage tolerance [5]. *E. coli *β clamp, like other DnaN family members, contains a hydrophobic cleft positioned near the C-terminus of each protomer that interacts competitively with a conserved clamp-binding motif (CBM) sequence present in most, if not all partners (see Figure 1 &2; [8]). Since the β clamp functions as a homodimer, it contains two such clefts, suggesting it may simultaneously manage the actions of two different partner proteins on DNA by acting as a molecular 'toolbelt.' In this model, each partner is bound to a different cleft in the clamp (see Figure 1A; [9-11]). Consistent with this model, a single cleft in the clamp is sufficient to support assembly of the clamp onto DNA, as well as processive replication by the replicative *E. coli *Pol (Pol III) using an *in vitro *system reconstituted with purified components, suggesting the other cleft is available for physical interaction with a second partner protein [12,13].

Although the CBM-clamp cleft interaction is essential for biological function of all known clamp partners examined to date, it is becoming increasingly evident that partners make functionally important contacts with non-cleft surfaces of the clamp as well (reviewed in [5]). For example, residues E93 and L98 of the clamp, which are located on the rim, interact with TLS Pols IV and V (see Figure 2C; [13-16]). Using a heterodimeric clamp protein (β^C^/β^+^) bearing a mutant protomer lacking a functional cleft (β^C^) in complex with a wild-type protomer (β^+^), we recently determined that a single cleft, together with the adjacent rim contact were both required for Pol IV to switch with a stalled Pol III *in vitro *(see Figure 1B; [13]). These results suggest a single clamp may utilize a combination of cleft and non-cleft surfaces to simultaneously manage the actions of more than two partners on DNA [5,13].

Besides their roles in TLS, Pols IV and V additionally act in a primitive DNA damage checkpoint control [17-19]. Pol II is also suggested to act in a checkpoint [19]. Interactions of these Pols with components of the replication machinery, particularly the β clamp, are suggested to slow or even arrest DNA replication following replication blocking damage to allow time for accurate repair (see Figure 1; [18-20]). However, the mechanistic relationship between the proposed checkpoint and Pol switching is presently unknown.

In addition to its protein partners, the clamp also interacts with the DNA template that it encircles [21,22]. Clamp-DNA interactions involve the cleft, as well as two additional clamp surfaces, one of which (residues H148-R152) also interacts with both Pol II and Pol IV [21]. Taken together, these results suggest that the clamp may play a direct role in physically sensing damaged DNA, and upon doing so, alter the way in which it interacts with the DNA template to enable recruitment of one or more DNA repair proteins, and/or specialized Pols to enable a series of concerted switches designed to coordinate replication with DNA repair and TLS [21].

Our current appreciation of clamp functionality is based largely on results of *in vitro *biochemical assays (reviewed in [5,7,23]). For example, recent *in vitro *experiments exploiting heterodimeric clamps comprised of two distinct β protomers revealed that a single cleft of the clamp was both necessary and sufficient for supporting a switch between Pol IV and a stalled Pol III [5,12,13]. However, many questions concerning the biological significance of this as well as other current models remains largely untested, due to the lack of a method for analyzing defined heterodimeric clamp proteins *in vivo*. As part of an effort to address this deficiency, we engineered a synthetic *dnaN *gene expressing tandem clamp protomers fused head-to-tail by a short amino acid linker bearing a His_6 _tag. Based on Western blot analysis using both anti-beta clamp and anti-Penta·His (Qiagen) antibodies, the linker sequence was susceptible to a significant level of proteolysis *in vivo *(J.M. Duzen and M.D. Sutton, unpublished results). We therefore pursued a separate strategy that exploited an inactive, mutant form of the β clamp lacking a functional cleft (β^C^) that we determined to be capable of complementing the temperature sensitive growth phenotype of the *dnaN159 *strain to provide a defined population of β^C^/β159 heterodimeric clamp protein for genetic analysis. Using this system, we tested several critical predictions of the models discussed above and summarized in Figure 1. Our findings, discussed below, suggest that a single cleft in the *E. coli *β clamp protein is sufficient to support cell viability, as well as manage the actions of Pols III, IV, and V during DNA replication and TLS *in vivo*.

## Results & Discussion

### β^C ^complements the temperature sensitive growth phenotype of the *dnaN159 *strain provided that Pol IV is inactivated

**Figure 3:**
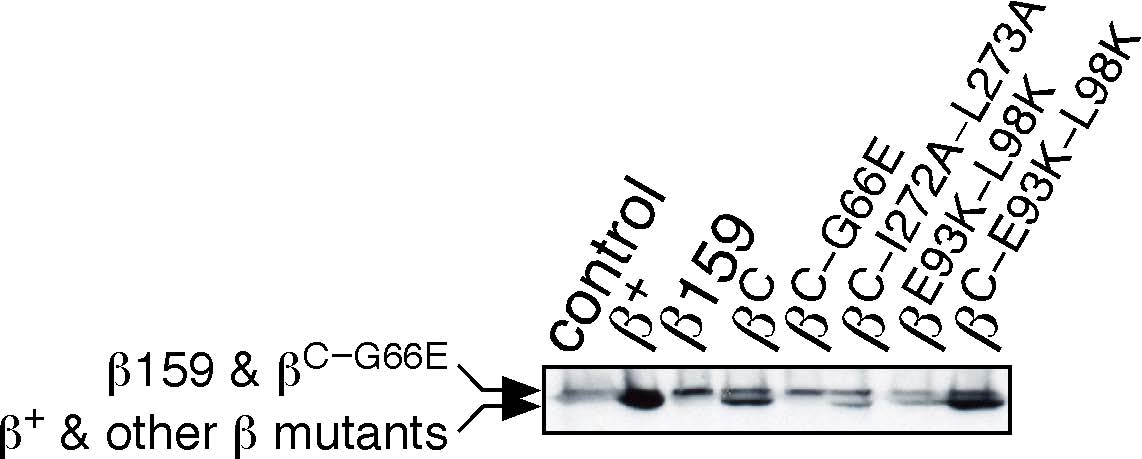
**Steady-state levels of mutant β clamp proteins**. Steady-state levels of the different clamp proteins were examined as described in *'METHODS.' *As noted in *'METHODS,' *we previously determined the number of clamp proteins expressed in *E. coli *MS101 bearing either pWSK29, or pWSK29-derived plasmids expressing different clamp proteins [27]. Note that both β159 and β^C-G66E ^possess altered mobility in SDS-PAGE relative to the wild-type clamp, or the other mutants, due to the G66E substitution [24].

β^C ^lacks a functional cleft due to deletion of the C-terminal five residues (see Figure 2), rendering it completely inactive for loading onto DNA, as well as for supporting Pol III replication [12]. In stark contrast, a heterodimeric form of the clamp bearing a recombinant β^C ^protomer in complex with a wild-type protomer (β^C^/β^+^) was recently determined to be indistinguishable from the wild-type clamp with respect to its ability to be loaded onto primed DNA, and stimulate processive DNA synthesis by Pol III *in vitro *[12,13]. The β^C^/β^+ ^heterodimer was also comparable to the wild-type clamp with respect to coordinating a switch between Pol IV and a stalled Pol III *in vitro *[13]. Based on these findings, we hypothesized that if a clamp bearing a single cleft was competent for supporting *E. coli *viability, then β^C ^might complement the temperature sensitive growth phenotype of the *dnaN159 *strain *via *formation of a functional β^C^/β159 heterodimeric clamp protein *in vivo *(see Figure 2B). The *dnaN159 *allele expresses a mutant clamp bearing G66E and G174A substitutions (β159; see Figure 2), and strains bearing this allele are unable to grow at temperatures above 37°C [24]. Of importance to the work discussed herein, β159 does not undergo detectable proteolysis during incubation at elevated temperatures (see Figure 3; [24-26]), and was capable of forming heterodimers with either the wild-type or a mutant β clamp protein bearing alanine substitutions of residues 148-152 *in vivo *[21].

**Figure 4:**
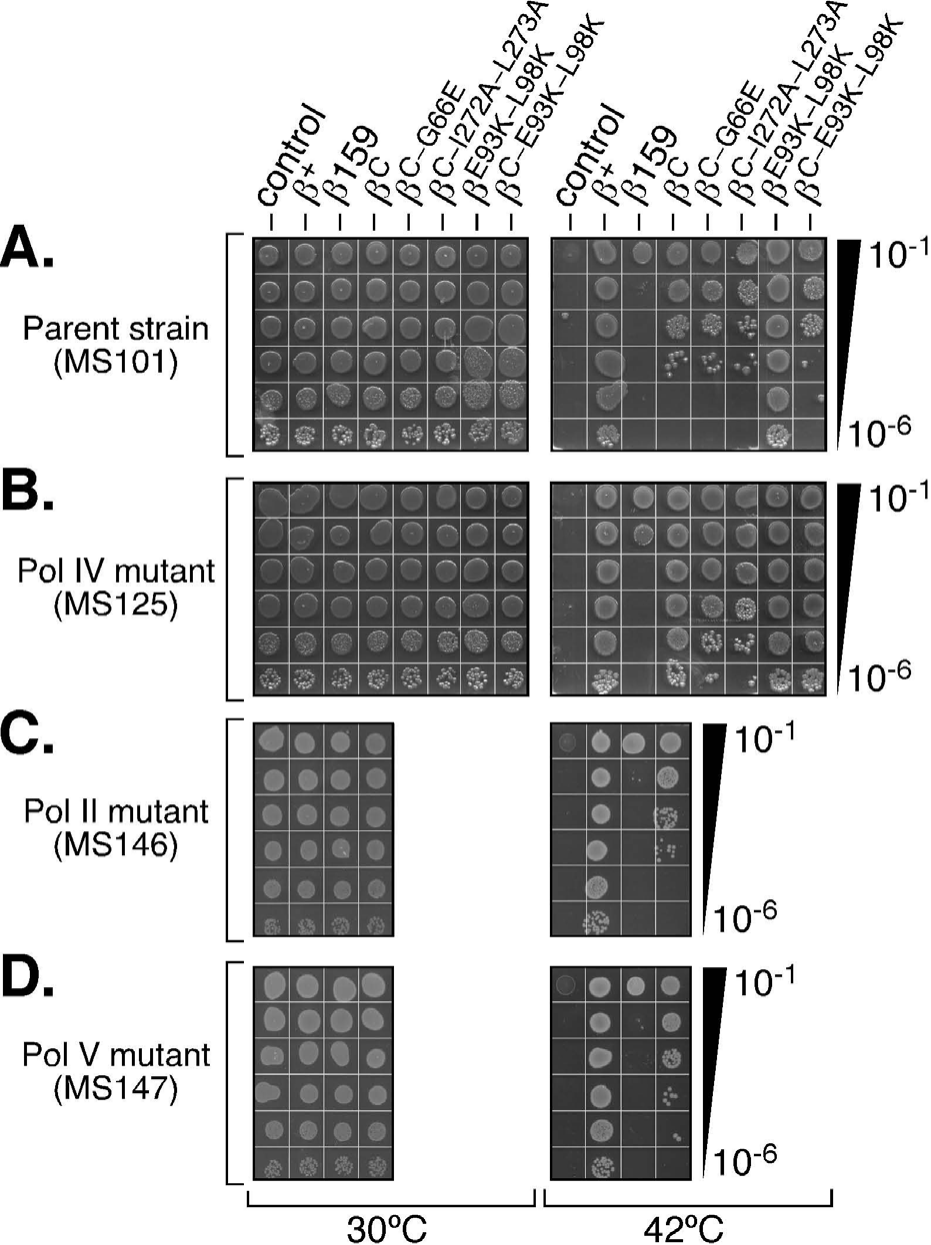
**Ability of β^C ^to complement the temperature-sensitive growth phenotype of the *dnaN159 *strain**. Serial dilutions of strains MS101 (*dnaN159*), MS125 [*dnaN159 *Δ(*dinB-yafN*)::*kan*], MS146 [*dnaN159 *Δ(*araD-polB*):: Ω], and MS147 (*dnaN159 *Δ*umuDC595*::*cat*) bearing plasmids directing physiological levels of expression of the indicated clamp proteins were spotted onto Luria-Bertani agar plates supplemented with Amp and IPTG. Plates were photographed following overnight incubation at 30° or 42°C, as indicated. At least two independent transformants for each strain were examined. Representative results are shown. 'Control' refers to the strain bearing the empty pWSK29 plasmid. See Table 1 for details concerning the *E. coli *strains and plasmids used.

We initiated these studies by first asking whether physiological levels of the wild-type, β159, or β^C ^clamp proteins, when expressed separately from a low-copy-number plasmid (Figure 3), were capable of complementing the temperature sensitive growth phenotype of the *dnaN159 *strain. Consistent with previous reports [26,27], strain MS101 bearing either the empty plasmid control (pWSK29), or the β159-expressing plasmid (pJD109), grew at 30°C, but not at 42°C (Figure 4). In contrast, this same strain containing a plasmid expressing the wild-type clamp (pJD100) grew equally well at both 30° and 42°C, indicating that β^+ ^fully complemented the temperature sensitivity of the *dnaN159 *strain. Despite the fact that a single cleft in the clamp was sufficient for supporting both clamp loading as well as Pol III function *in vitro *[12], β^C ^was unable to complement temperature sensitivity of the *dnaN159 *strain (Figure 4). Based on Western blotting, β^C ^was expressed at physiological levels (Figure 3), implying that its inability to complement the *dnaN159 *strain was due to a functional defect.

We have previously described circumstances under which Pol II, Pol IV and/or Pol V are capable of interfering with viability of the *dnaN159 *strain [21,24,26,28,29]. In light of these findings, we hypothesized that the inability of β^C ^to complement the *dnaN159 *strain may be due to the action of one or more specialized Pols. In order to distinguish between a model in which the inability of β^C ^to complement temperature sensitive growth of the *dnaN159 *strain was attributable to a requirement of both clamp clefts for *E. coli *viability, and a model in which a single cleft in the clamp is sufficient to support viability, but one or more TLS Pols was acting to impair function of the β^C^/β159 heterodimer at 42°C, we asked whether β^C ^could support temperature resistant growth of a *dnaN159 *strain lacking either Pol II [Δ(*araD-polB*):: Ω], Pol IV [Δ(*dinB-yafN*)::*kan*], or Pol V (Δ*umuDC596*::*ermGT*) function. As shown in Figure 4 β^C ^was able to fully complement temperature sensitive growth of the *dnaN159 *strain lacking Pol IV function [Δ(*dinB-yafN*)::*kan*]. In contrast, β^C ^was unable to complement growth of the *dnaN159 *strain lacking Pol II (MS146) or Pol V (MS147). Thus, Pol IV may outcompete Pol III for interaction with the β^C^/β159 clamp to impair growth. Alternatively, the β^C^/β159 clamp may be sensitized to the checkpoint function of Pol IV. Regardless of the mechanism(s) by which Pol IV acts to impair growth of the β^C^-expressing strain, the fact that β^C^, on its own, is non-functional [12], together with our finding that β159 cannot support cell viability at 42°C, even when expressed at an elevated level (Figure 3 &4), argues strongly that growth at 42°C of the *dnaN159 *strain relies entirely on the ability of a temperature resistant β^C^/β159 heterodimeric clamp to fulfill all essential clamp functions.

### Disruption of the Pol IV-clamp rim contact in β^C ^fails to alleviate the lethal effect of Pol IV

**Figure 5:**
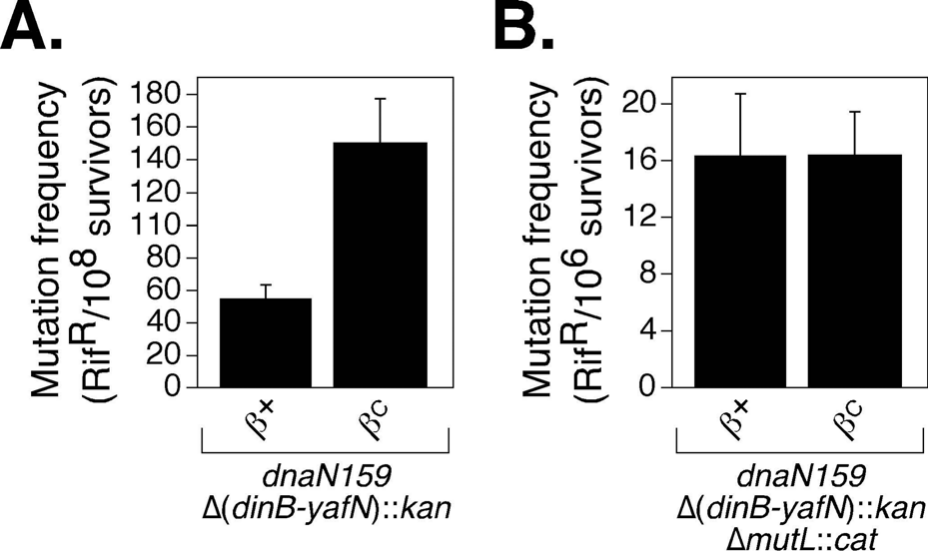
**Ability of β^C ^to support Pol V-dependent UV-induced mutagenesis *in vivo***. UV-induced mutagenesis was measured using isogenic **(A) ***mutL*^+ ^(MS125) and **(B) **Δ*mutL*::*cat *(MS148) *dnaN159 *Δ(*dinB-yafN*)::*kan *strains expressing physiological levels of either β^+ ^(pJD100) or β^C ^(pMDS110), as described in *'METHODS.' *Results shown are the average of at least 4 determinations. Error bars represent the standard deviation.

The little finger domain of Pol IV (Pol IV^LF^) interacts with the clamp by bridging the dimer interface [14]. In solution, and in the absence of DNA, two Pol IV molecules simultaneously contact a single clamp, with each Pol IV contacting the rim of one protomer, and the cleft of the adjacent clamp protomer (see Figure 2C; [13-15]). As discussed above, the cleft contact is required for stimulation of Pol IV replication [13,30], while the rim contact is required for Pol IV to undergo a switch with a stalled Pol III [13]. We therefore asked whether mutating critical residues in the rim in β^C^, which is adjacent to the cleft in β159 (see Figure 2 panels D & C), alleviated the need to inactivate Pol IV in order for β^C ^to complement temperature sensitivity of the *dnaN159 *strain. The combination of the E93K and L98K mutations in the clamp was previously demonstrated to severely impair interaction of Pol IV with the rim [13]. A clamp mutant bearing only E93K-L98K substitutions (β^E93K-L98K^) was expressed at a level comparable to the wild-type clamp (Figure 3). Moreover, the β^E93K-L98K ^mutant clamp fully supported growth of the *dnaN159 *strain at 42°C, indicating that these residues were dispensable for essential clamp function(s) *in vivo *(Figure 4). In striking contrast, β^C-E93K-L98K ^was unable to complement the *dnaN159 *strain, unless Pol IV was inactivated (Figure 4), despite the fact that the mutant clamp was expressed at physiological levels (Figure 3). Taken together, these results indicate that the ability of Pol IV to impair growth of the β^C^-expressing strain is not the result of Pol IV gaining access to the cleft in β159 by first binding to the rim of β^C ^in a manner similar to that by which Pol IV switches with a stalled Pol III (see Figure 1B; [5,13]). It is possible that Pol IV outcompetes Pol III for binding to the cleft of the β159 protomer, independently of the rim contact, leading to cell death at 42°C. Alternatively, binding of multiple Pol IV molecules to some combination of rim and cleft regions of a single β^C^/β159 clamp may act to preclude access and/or function of Pol III, possibly *via *a DNA damage checkpoint response. The affinity of Pol IV for the rim of the clamp is on the order of ~1 μM [13], which is intermediate to the SOS-repressed (~300 nM) and SOS-induced levels (~3,300 nM) of Pol IV [5,31], providing support for these models.

### β^C ^complements the temperature sensitive growth phenotype of the *dnaN159 *strain *via *a β^C^/β159 heterodimer

Results discussed above suggest that growth of the *dnaN159 *strain at 42°C relies on a β^C^/β159 heterodimer. We pursued two parallel strategies to test this hypothesis. In our first approach, we sought to purify for subsequent biochemical analysis a recombinant form of the β^C^/β159 heterodimer using an established protocol [12]. Although this recombinant clamp protein was expressed in a soluble form, it became poorly soluble when purified to homogeneity, making it impossible to rigorously establish its purity as a heterodimer, or to accurately measure its ability to support processive DNA replication *in vitro *(data not shown).

In a parallel strategy, we employed a genetic approach to determine whether a β^C^/β159 heterodimer supported growth of the *dnaN159 *strain. Residues I272 and L273 of β map to the dimer interface, and their substitution with alanine in the wild-type clamp leads to its monomerization *in vitro *[32]. We hypothesized that substitution of these residues in β^C ^(β^C-I272A-L273A^) would destabilize the β^C^/β159 heterodimer *in vivo *(see Figure 2D), thereby impairing growth at 42°C. Consistent with our hypothesis, β^C-I272A-L273A ^was unable to fully complement temperature sensitivity of the *dnaN159 *strain, irrespective of Pol IV function (Figure 4). Our finding that β^C-I272A-L273A ^was expressed at near physiological levels (Figure 3) suggests that its inability to complement temperature sensitivity results from a functional defect. Efforts to clone a β^I272A-L273A^-expressing plasmid to measure its ability to complement the *dnaN159 *strain as a negative control were unsuccessful, suggesting that a monomeric form of the clamp bearing a functional cleft exerts a dominant-negative phenotype *in vivo*.

As part of this same strategy, we also substituted residue G66 of β^C ^with glutamic acid (β^C-G66E^). Temperature sensitivity of β159 requires both the G66E and G174A substitutions [24]. We hypothesized that introduction of the G66E substitution into β^C ^(β^C-G66E^) would effectively mimic its effect in β159, rendering both the β^C-G66E ^mutant, as well as the β^C-G66E^/β159 heterodimer thermolabile. Consistent with this hypothesis, β^C-G66E ^was unable to fully complement growth of the *dnaN159 *strain (Figure 4). Based on Western blotting (Figure 3), β^C-G66E ^was expressed at physiological levels (note that the G66E substitution slows mobility of clamp in SDS-PAGE [24]). Thus, taken together, results discussed above suggest that a β^C^/β159 heterodimer supports growth of the *dnaN159 *strain at 42°C, suggesting that a single cleft in the clamp is capable of supporting all essential clamp functions *in vivo*.

### β^C ^supports DNA damage-induced mutagenesis in the *dnaN159 *strain

Our finding that a β^C^/β159 heterodimer supports growth of the *dnaN159 *strain at 42°C provided us with a tractable system to determine whether a clamp bearing a single functional cleft was capable of coordinating the actions of Pol III and TLS Pols following DNA damage. Since growth of the β^C ^strain at 42°C required that the gene for Pol IV be deleted, we were unable to analyze Pol IV function. However, we were able to analyze Pol V (*umuDC*) function. For this, we cultured the *dnaN159 *Δ(*dinB-yafN*)::*kan *strain expressing either the wild-type or β^C ^clamp from a plasmid in liquid broth at 42°C. Exponential phase cultures were either irradiated with 254 nm ultraviolet light (UV), or mock irradiated, and appropriate dilutions of each culture were plated to determine the frequency of spontaneous and UV-induced Rif^R^. Both the β^+ ^(4.4 ± 1.7 × 10^-9^) and β^C ^(4.8 ± 1.7 × 10^-9^) strains displayed spontaneous mutation frequencies comparable to those reported for similar *E. coli *strains [29,33]. DNA mismatch repair (MMR) function is required for correcting replication errors, ensuring a low (*e.g*., normal) spontaneous mutation frequency [1]. Inasmuch as interaction of both MutS and MutL with the clamp is required for MMR *in vivo *[34], these findings suggest that a single cleft in the clamp is capable of supporting MMR, and coordinating it with replication. In contrast to their spontaneous mutation frequencies, the frequency of UV-induced mutagenesis was ~2.5-fold higher in the β^C^-expressing strain compared to the wild-type control (Figure 5A), suggesting that the β^C^/β159 heterodimer may be impaired for proper coordination of Pol III and Pol V, resulting in more frequent access of Pol V to the replication fork following UV irradiation. Although we cannot rule out the possibility that partially functional β159 homodimers persist in the *dnaN159 *strain, we previously determined that β159 homodimers were impaired for Pol V-dependent UV mutagenesis at 37°C [24,27]. Taken together, these results argue that the β^C^/β159 heterodimer supports Pol V-dependent mutagenesis at 42°C.

In addition to correcting replication errors catalyzed by Pol III, MMR also acts to correct errors catalyzed by Pol V during TLS, including misinsertions opposite thymine-thymine dimers [35], as well misinsertions opposite undamaged bases adjacent to UV adducts which, if left uncorrected, result in 'hitchhiker' mutations [36]. Since the mechanism by which MMR catalyzes repair during TLS may differ from that during Pol III replication, we measured the frequency of UV-induced mutagenesis in the MMR-deficient Δ*mutL*::*cat *strain background (MS148). As summarized in Figure 5B, frequencies of UV-induced mutagenesis in the β^+ ^and β^C ^strains were comparable, suggesting that β^C^/β159 was able to properly manage the actions of Pol III and Pol V *in vivo*. The spontaneous mutation frequency of the β^C ^strain was elevated ~2.5-fold relative to the β^+ ^strain (6.9 × 10^-6 ^compared to 2.5 × 10^-6^), suggesting that one or more aspects of DNA replication and/or accurate repair were modestly affected by the β^C^/β159 clamp. Interestingly, a different *dnaN159 *Δ*mutL*::*cat *strain displayed a similarly elevated spontaneous mutation frequency at the permissive temperature of 30°C [33], suggesting that this phenotype was due to the β159 protomer. Taken together, results discussed above suggest that although a single cleft in the β clamp is sufficient to coordinate the actions of Pol III and Pol V *in vivo *(see Figure 1B), both clefts are required for proper MMR function during TLS.

## Conclusions

Results summarized in this report indicate that β^C ^complements both the temperature sensitivity and Pol V-dependent UV-induced mutator defect of the *dnaN159 *strain, provided that Pol IV is inactivated (Figure 4 &5). Taken together, these findings suggest that a single cleft in the clamp is sufficient to support all essential clamp functions *in vivo*, and provide additional support for our model that a single cleft is sufficient to coordinately manage the actions of multiple clamp partners on DNA (see Figure 1B; [5,13]). It is presently unclear why Pol IV function prevents β^C ^from complementing the *dnaN159 *strain. It is possible that Pol IV associates with surfaces in addition to the rim and cleft to somehow impair growth, possibly as part of a DNA damage checkpoint control (see Figure 1; [18,19]). Alternatively, Pol IV may simply out compete Pol III for access to the β^C^/β159 clamp on leading and/or lagging strand. Other scenarios are also possible. Regardless of the mechanism(s), the fact that Pol IV impairs growth of the β^C ^strain suggests that a single cleft on the clamp is also sufficient to support Pol IV function(s) *in vivo*. An obvious limitation of the method used to generate heterodimeric clamps *in vivo *is the possibility that partially functional β159 homodimers persist. Rational design of novel mutant clamp proteins bearing site-specific mutations at the dimer interface that impair homodimerization while simultaneously enabling dimerization *in trans *(*e.g*., heterodimers) would circumvent this issue, and would provide a powerful approach for dissecting mechanisms by which the clamp manages events at a replication fork *in vivo*. Finally, although not addressed in this study, proteins in addition to clamp, as well as the DNA template itself contribute to Pol switching (reviewed in [5]). Defining the respective contributions of these non-clamp factors in this multifaceted process will be made simpler once the roles played by clamp are defined in molecular terms.

## Methods

### Bacteriological techniques

Isogenic *E. coli *strains and plasmid DNAs utilized in this work are described in Table 1. Strain DH5α was used as host for cloning plasmids. Strains MS146, MS147, and MS148 were constructed by generalized transduction using P1*vir *[37]. The presence of the Δ(*araD-polB*):: Ω allele was confirmed by diagnostic PCR, as described previously [26]. Strains were routinely cultured in Luria-Bertani medium (LB; 10 g/l tryptone, 5 g/l yeast extract, 10 g/l sodium chloride; [37]). Strains bearing plasmids were grown in medium containing ampicillin (Amp) at a final concentration of 150 μg/ml. When noted, IPTG was added at a final concentration of 50 μM to induce expression of physiological levels of the plasmid-encoded clamp protein [27]. At least two independent plasmid clones were separately transformed into each strain, and at least two independent transformants of each plasmid/strain were used for each experiment.

**Table 1:** E. coli strains, plasmid DNAs, and synthetic oligonucleotides used in this study.

*E. coli *strains:		
**Strain**	**Relevant genotype**	**Source**

DH5α	*endA1 hsdR17*(r_K_^- ^m_K_^+^) *glnV44 thi-1 recA1 gyrA96 relA1 deoR nupG *Δ(*lacZYA-argF*)*U169 *(φ80*dlacZ*ΔM15)	Invitrogen

MS101	*thr-1 araD139 *Δ(*gpt-proA*)*62 lacY1 tsx-33 supE44 galK2 hisG4*(Oc) *rpsL31 xyl-5 mtl-1 argE3*(Oc) *thi-1 sulA211 dnaN159*(Ts) *tnaA300*::Tn*10*	[26]

MS146	MS101 with Δ(*araD-polB*):: Ω	This work

MS125	MS101 with Δ(*dinB-yafN*)::*kan*	[28]

MS147	MS101 with Δ*umuDC596*::*ermGT*	This work

MS148	MS101 with Δ(*dinB-yafN*)::*kan *Δ*mutL*::*cat*	This work

**Plasmid DNAs:**		

**Plasmid**	**Relevant characteristics**	**Source**

pWSK29	Amp^R^; pSC101 origin; low copy number general cloning vector	[39]

pJD100	Amp^R^; pWSK29 containing the *dnaN^+ ^*(β^+^) gene under the control of its native promoters	[26]

pJD109	Amp^R^; pJD100 bearing *dnaN159 *(G66E and G174A substitutions; β159)	[27]

pMDS110	Amp^R^; pJD100 bearing *dnaN *lacking residues 362-366 (*dnaN^C^*; β^C^)	This work

pMDS111	Amp^R^; pJD100 bearing *dnaN *lacking residues 362-366 and containing a G66E substitution (*dnaN^C-G66E^*; β^C-G66E^)	This work

pMDS112	Amp^R^; pJD100 bearing *dnaN *lacking residues 362-366 and containing I272A and L273A substitutions (*dnaN^C-I272A-L273A^*; β^C-I272A-L273A^)	This work

pMDS113	Amp^R^; pJD100 bearing *dnaN *containing E93K and L98K substitutions (*dnaN^E93K-L98K^*; β^E93K-L98K^)	This work

pMDS114	Amp^R^; pJD100 bearing *dnaN *lacking residues 362-366 and containing E93K and L98K substitutions (*dnaN^C-E93K-L98K^*; β^C-E93K-L98K^)	This work

**Oligonucleotides:**		

**Name**	**Nucleotide sequence (5'→3')**	

Δ362-366-T	GGCTTATGTTGTCTAATGAATGAGACTG	

Δ362-366-B	CAGTCTCATTCATTAGACAACATAAGCC	

G66E-T	CAGCCACACGAGCCAGAAGCGACGACCGTTCCGG	

G66E-B	CCGGAACGGTCGTCGCTTCTGGCTCGTGTGGCTG	

I272A-L273A-T	GTTTGCTCGCGCGGCGGCTGCCTCTAACGAGAAATTCCG	

I272A-L273A-B	CGGAATTTCTCGTTAGAGGCAGCCGCCGCGCGAGCAAAC	

E93K-L98K-T	CGTGCAGCTGAAAGGTGAACGGATGAAAGTACGCTCCGG	

E93K-L98K-B	CCGGAGCGTACTTTCATCCGTTCACCTTTCAGCTGCACG	

### Site-directed mutagenesis

Site-directed mutagenesis was performed using the Quick-Change mutagenesis kit (Stratagene). Primers employed in mutagenesis were synthesized by Sigma-Genosys, and their sequences are listed in Table 1. Plasmid pJD100 (*dnaN^+^*), or pMDS110 (*dnaN^C^*; see Table 1), served as template for PCR reactions. PCR amplification was for 18 cycles of 95°C for 30 s, 55°C for 1 min, 68°C for 12 min. After PCR, reactions (50 μl) were treated with *Dpn*I (10 U) for 1 h to digest the parental template DNA prior to transforming it into chemically competent DH5α. Transformants were selected by virtue of their resistance to Amp. Plasmid clones were individually purified using the Qiagen Spin Prep kit, and screened by restriction analysis prior to determining the nucleotide sequence of two independent clones for each (Roswell Park Cancer Center Biopolymer Facility).

### Western blotting

Western blot analysis was performed as described previously [27] using cultures of MS101 bearing the indicated plasmids grown at 30°C (the permissive temperature for the pWSK29 [control] and pJD109 [*dnaN159*] transformants) in LB medium supplemented with Amp and IPTG. When cells reached exponential phase (OD_595 _~0.5), a volume of culture equivalent to 1 ml of OD_595 _= 1.0 was harvested by centrifugation. Cell pellets were washed once with 0.8% saline prior to being resuspended in SDS-PAGE loading buffer (50 mM Tris-HCl [pH 6.8], 25 mM dithiothreitol, 2% SDS, 0.2% bromophenyl blue, and 10% glycerol) at a density of ~10^7 ^cells/μl. Ten μl (~10^8 ^cells) of each sample was electrophoresed through a 12% SDS-PAGE, transferred to PVDF, blotted with rabbit polyclonal anti-β antibodies, and immuno-reactive material was detected using the Super Signal Western Dura Extended Chemiluminescence substrate (Pierce) as described previously [27]. Based on quantitative Western blot analysis, strain MS101 (*dnaN159*) expressed 281 ± 90 clamps/cell (as dimer), while MS101 bearing pJD100 (β^+^) expressed 1,144 ± 532 clamps/cell [27].

### UV-induced mutagenesis

UV-induced mutagenesis was performed as described previously [27]. Briefly, cultures were grown to exponential phase (OD_595 _~0.5) at 42°C in liquid LB medium supplemented with Amp and IPTG, at which point cells were harvested by centrifugation, washed twice with 0.8% saline before being resuspended in saline, and either UV irradiated (25 J/m^2^) using a germicidal lamp (254 nM, GE Healthcare) or mock irradiated. Following irradiation, cells were allowed to recover overnight at 42°C in liquid LB medium supplemented with Amp prior to plating appropriate dilutions onto LB agar plates containing Amp with or without 100 μg/ml Rif. Mutation frequency was calculated by dividing the number of Rif^R ^CFU/ml by the total number of viable cells/ml. UV-induced mutation frequency is expressed as the frequency of Rif^R ^observed following exposure to UV minus the spontaneous mutation frequency observed for the mock irradiated control.

## List of Abbreviations

Pol: DNA polymerase; TLS: translesion DNA synthesis; CBM: clamp-binding motif; UV: ultraviolet light; MMR: mismatch repair; LB: Luria-Bertani; Amp: ampicillin; Amp^R^: ampicillin resistance; Rif: rifampicin; Rif^R^: rifampicin resistant.

## Authors' contributions

MDS conceived the project, designed the experiments, and wrote the paper. All three authors contributed to performing the experiments, and each read and approved the final manuscript.
